# Measurement Properties of the Online EuroQol-5D-Youth Instrument in Children and Adolescents With Type 1 Diabetes Mellitus: Questionnaire Study

**DOI:** 10.2196/14947

**Published:** 2019-11-12

**Authors:** Karina Mayoral, Luis Rajmil, Marta Murillo, Olatz Garin, Angels Pont, Jordi Alonso, Joan Bel, Jacobo Perez, Raquel Corripio, Gemma Carreras, Javier Herrero, Jose-Maria Mengibar, Dolors Rodriguez-Arjona, Ulrike Ravens-Sieberer, Hein Raat, Vicky Serra-Sutton, Montse Ferrer

**Affiliations:** 1 Health Services Research Group Hospital del Mar Medical Research Institute Barcelona Spain; 2 Department of Paediatrics Obstetrics and Gynaecology and Preventive Medicine Universitat Autònoma de Barcelona Barcelona Spain; 3 Centro de Investigación Biomédica en Red de Epidemiología y Salud Pública Madrid Spain; 4 Agency for Health Quality & Assessment of Catalonia Barcelona Spain; 5 Pediatric Service Department of Pediatric Endocrinology University Hospital Germans Trias i Pujol Barcelona Spain; 6 Experimental and Health Sciences Pompeu Fabra University Barcelona Spain; 7 Department of Pediatric Endocrine Hospital of Sabadell Corporació Sanitària Parc Taulí Sabadell Spain; 8 University Institute Parc Taulí Universitat Autònoma de Barcelona Sabadell Spain; 9 Pediatric Endocrinology Hospital de la Santa Creu i Sant Pau Barcelona Spain; 10 Medicine Department Universitat Autònoma de Barcelona Barcelona Spain; 11 Corporació de Salut del Maresme i la Selva Hospital de Calella Calella Spain; 12 Corporació de Salut del Maresme i la Selva Hospital de Blanes Blanes Spain; 13 Department of Child and Adolescent Psychiatry, Psychotherapy, and Psychosomatics University Medical Center Hamburg - Eppendorf Hamburg Germany; 14 Department of Public Health Erasmus University Medical Center Rotterdam Rotterdam Netherlands

**Keywords:** health-related quality of life, type 1 diabetes, EQ-5D-Y, EuroQol, validity

## Abstract

**Background:**

The lack of continuity between health-related quality of life (HRQoL) instruments designed for children and adults hinders change analysis with a life course approach. To resolve this gap, EuroQol (EQ) developed the EQ-5D-Youth (EQ-5D-Y), derived from the EQ-5D for adults. Few studies have assessed the metric properties of EQ-5D-Y in children with specific chronic conditions, and none have done so for children with type I diabetes mellitus (T1DM).

**Objective:**

This study aimed to evaluate the acceptability, validity, reliability, and responsiveness of the EQ-5D-Y in children and adolescents with T1DM, when administered online.

**Methods:**

Participants with T1DM were consecutively recruited from July to December 2014, from a list of potential candidates aged 8-19 years, who attended outpatient pediatric endocrinology units. Before every quarterly routine visit, participants received an email/telephone reminder to complete the online version of two generic HRQoL questionnaires: EQ-5D-Y and KIDSCREEN-27. The EQ-5D-Y measures five dimensions, from which an equally weighted summary score was constructed (range: 0-100). Completion rate and distribution statistics were calculated. Construct validity was evaluated through known group comparisons based on general health, acute diabetic decompensations, mental health, family function, and a multitrait, multimethod matrix between EQ-5D-Y and KIDSCREEN by using Spearman correlations. Construct validity hypotheses were stated a priori. Reliability was assessed with the intraclass correlation coefficient and responsiveness by testing changes over time and calculating the effect size. Reliability and responsiveness were tested among the stable and improved subsamples defined by a KIDSCREEN-10 index change of <4.5 points or ≥4.5 points, respectively, from the first to the fourth visit.

**Results:**

Of the 136 participants, 119 (87.5%) responded to the EQ-5D-Y at the last visit. The dimensions that showed higher percentages of participants with problems were “having pain/discomfort” (34.6%) and “worried/sad/unhappy” (28.7%). The mean (SD) of the EQ-5D-Y summary score was 8.5 (10.9), with ceiling and floor effects of 50.7% and 0%, respectively. Statistically significant HRQoL differences between groups defined by their general health (excellent/very good and good/regular/bad) and mental health (Strengths and Difficulties Questionnaire score ≤15 and >16, respectively) were found in three EQ-5D-Y dimensions (“doing usual activities,” “having pain/discomfort,” and “feeling worried/sad/unhappy”), summary score (effect size for general health and mental health groups=0.7 and 1.5, respectively), and KIDSCREEN-10 index (effect size for general health and mental health groups=0.6 and 0.9, respectively). Significant differences in the EQ-5D-Y dimensions were also found according to acute diabetic decompensations in “looking after myself” (*P*=.005) and according to family function in “having pain/discomfort” (*P*=.03). Results of the multitrait, multimethod matrix confirmed three of the four relationships hypothesized as substantial (0.21, 0.58, 0.50, and 0.46). The EQ-5D-Y summary score presented an intraclass correlation coefficient of 0.83. Statistically significant change between visits was observed in the improved subsample, with an effect size of 0.7 (*P*<.001).

**Conclusions:**

These results support the use of the EQ-5D-Y administered online as an acceptable, valid, reliable, and responsive instrument for evaluating HRQoL in children and adolescents with T1DM.

## Introduction

Type 1 diabetes mellitus (T1DM) is one of the most common chronic childhood illnesses, affecting approximately 1 in every 400-600 children and adolescents [1]. Treatment includes a multifaceted regimen with daily subcutaneous insulin injections (or insulin infusion), blood glucose monitoring, carbohydrate counting, dietary plan, and physical activity [2]. Due to its complexity, T1DM management can be overwhelming even for the most competent patient [3]. Children might feel different from their peers because of their need for disruptive self-care activities [4] and the impact of T1DM on psychological aspects [5]. Health-related quality of life (HRQoL) captures the individual’s health perception, adding the patients’ perspective into T1DM clinical monitoring and research.

The lack of correspondence and continuity between HRQoL instruments designed for children and those designed for adults hinders the analysis of HRQoL changes using a life course approach. To solve this gap, in 2006, the EuroQol Group developed the EQ-5D-Youth (EQ-5D-Y) as a version of the EQ-5D for adults. The EQ-5D-Y was designed by adapting the EQ-5D dimensions to the requirements of HRQoL measurement in children and adolescents aged 8 years or older [6,7]. The main rationale of the new version was to enable young individuals to self-report their health [6,8]. A EQ-5D-Y proxy version was also developed [9] for children below 8 years of age. Given its generic and econometric nature, it allows comparison between different populations and conditions, and cost-utility analysis for economic evaluation. Another econometric instrument, the Health Utility Index [10], has a self-administered version for adults and adolescents and a proxy version for children (5-12 years of age), but its administration burden is substantially greater.

The EQ-5D-Y is a very short (2-3 minutes) and simple instrument to fill out. There are several studies assessing the metric properties of the EQ-5D-Y in the general population [11,12] or in school environments [13-16]. A Swedish study [12] suggested that the EQ-5D-Y was comprehensible, acceptable, and feasible for self-completion. A multicountry study supported its feasibility, reliability, and validity [11]. Furthermore, a Spanish study [16] compared the paper and Web-based versions of EQ-5D-Y, showing acceptable levels of agreement.

Only a few studies have assessed the metric properties of EQ-5D-Y in children with specific chronic conditions. A multicenter study among patients with cystic fibrosis [17] concluded that the EQ-5D-Y can be considered a valid instrument, which reflects the differences in health according to the progression of this life-long chronic disease. A comparison between the EQ-5D-Y and the Pediatric Asthma Quality of Life Questionnaire [18] supported EQ-5D-Y’s convergent validity for asthmatic children and adolescents. In addition, a study in youth with chronic kidney disease provided evidence of the EQ-5D-Y validity, as it discriminated among groups that differed with regard to the disease-related clinical burden [19]. Finally, we have only identified one descriptive study of HRQoL in children with T1DM [20] measured with the EQ-5D-Y, but without assessing its metric properties. Our aim was to evaluate acceptability, validity, reliability and responsiveness of the EQ-5D-Y administered online in children and adolescents with T1DM.

Data was obtained from a clinical trial on children and adolescents with T1DM, designed to evaluate the impact of routine use of HRQoL assessment in clinical practice by using the KIDSCREEN-27 as the primary outcome. Previous publications of this trial reported that the baseline HRQoL of children and adolescents with T1DM [21] was similar to that of the European population of the same age, but with slightly worse physical well-being, and that routine assessment and face-to-face patient-physician discussion of HRQoL results improved after a year of follow-up, especially psychological well-being and school environment [22].

## Methods

### Participants and Study Design

Subjects were consecutively recruited from July to December 2014, from a list of 205 potential candidates with T1DM, aged between 8 and 19 years, attending outpatient pediatric endocrinology units of 5 hospitals. Exclusion criteria were as follows: T1DM diagnosed within the last 6 months and presence of cognitive problems that prevented comprehension of the questionnaires. Seven pediatricians were randomly assigned to either the intervention or control group (four and three pediatricians, respectively).

Families of children that fulfilled the inclusion criteria were informed about the project, and those who agreed to participate received a reminder by email and then by telephone, if necessary, before every quarterly routine visit to complete the online questionnaires within the 48 hours prior to the visit (for children with limited access to internet, a laptop was available at the doctor’s office).

The intervention involved a discussion of the HRQoL scores between the doctor and the participant at each routine visit. The KIDSCREEN-27 and EQ-5D-Y results were displayed with different colors, similar to a traffic light, marking green for good, yellow for medium, and red for poor outcomes. Evolutionary results were displayed in the second, third, and fourth visits, to show a comparison with the previous results. At each visit, pediatricians invited the patients to comment and discuss the results, identifying dimensions with low value scores, exploring possible solutions and actions, and coming back to these actions over time with advice. Before starting the study, the pediatricians in this group had received standardized training in the use and interpretation of HRQoL questionnaires.

The control group also completed the questionnaires online. However, the questionnaires completed by the control group 48 h before each visit were about physical activity and diet, and the HRQoL questionnaires were completed only before the first and fourth visits. During the consultation, the patients received usual care without commenting on the results of the questionnaires (physicians did not receive any instruction on clinical management).

### Study Variables

Information collected from clinical records included age, gender, weight, height, and glycated hemoglobin (HbA_1c_) levels. Acute diabetic decompensations during the previous 3 months were registered: hypoglycemia (<60 mg/dL) with decreased level of consciousness, requiring glucagon or the help of others to recover, and hyperglycemia (>400-450 mg/dL), which required intervention of professionals or presented with diabetic ketoacidosis.

HRQoL evaluation consisted of the administration of the Spanish online version of the KIDSCREEN-27 and the EQ-5D-Youth (EQ-5D-Y). The descriptive system of the EQ-5D-Y [6] consists of five dimensions (“mobility,” “looking after myself,” “doing usual activities,” “having pain/discomfort,” and “feeling worried/sad/unhappy”) with three-level Likert scale responses (no problems, moderate problems, and serious problems). It includes also a visual analogue scale (EQ-VAS) on the general health status rated from 0 (worst health status) to 100 (best health status possible). The time frame for both dimensions and EQ-VAS is “today.” As no preference weights were available for the EQ-5D-Y to estimate utilities [23], an equally weighted summary score of the five dimensions was calculated [24-26], ranging from 0 (no problem in any dimension) to 100 (serious problems in all dimensions).

The KIDSCREEN-27 [27] contains 5 dimensions: physical well-being (5 items), psychological well-being (7 items), autonomy and relationships with parents (7 items), social support and relationship with friends (4 items), and school environment (4 items). Responses were categorized into five-option Likert scales that assess the frequency or intensity of the attribute, with a recall period of 1 week in most questions. Scores were calculated following developers’ recommendations [27] for the KIDSCREEN-27 dimensions and for the KIDSCREEN-10 index, constructed with 10 items that sufficiently represent the longer KIDSCREEN profiles. The KIDSCREEN scores are standardized to a mean of 50 and a SD of 10, from a reference sample of 22,000 European children and adolescents [28].

In addition to HRQoL, children’s mental health status and family function were assessed. The Strengths and Difficulties Questionnaire (SDQ) [29] consists of 25 items measuring a range of mental health symptoms including conduct problems, hyperactivity-inattention, emotional symptoms and peer problems, and prosocial behaviors. All items are scored on a 3-point scale (0=not true, 1=somewhat true, and 2=certainly true). Items are summed to give a total difficulties score ranging from 0 (no problems) to 40 (maximum problems). The Spanish version of the SDQ has shown to be reliable and valid [30]. The family function questionnaire [31,32] was designed to measure the patient’s satisfaction with the support from the family through five items: Adaptation, Partnership, Growth, Affection, and Resolve (APGAR). These items have three response options (from almost always to hardly ever), and the total score ranges from 0 to 10 (low to high satisfaction).

### Ethics Considerations

The study was approved by the ethics committee of participant hospitals in accordance with national and international guidelines (code of ethics, Helsinki Declaration) as well as legislation on data confidentiality (Organic Law 15/1999 of December 13 Data Protection character staff). Signed consent to participate was requested from parents and children over 12 years. The collection and transfer of data were carried out according to strict security and data encryption.

### Analytical Strategy

Considering the 136 patients included in the T1DM clinical trial, this sample size gave a statistical power of 0.80 to detect a moderate-to-large difference of 0.65 SDs in the EQ-5D-Y summary score between unequally distributed known groups (80% and 20% of participants in each category) using a two-sided test with type I error of 5%. Statistical power was calculated using published formulas [33].

Characteristics of the sample were described by calculating percentages, or means and SDs, according to the type of variable. To evaluate the acceptability of the EQ-5D-Y, we calculated the completion rate and the distribution of the response options in each dimension. Distribution of the EQ-5D-Y summary score, EQ-VAS, and the KIDSCREEN-10 index was described by calculating the observed range, floor, and ceiling effects (proportion of participants with the worst and best possible score, respectively) and statistics of central tendency and dispersion.

Construct validity was assessed by applying two different approaches: (1) comparison of known groups and (2) the multitrait, multimethod matrix with data from the first visit. Known groups were defined according to the general health (through a question from KIDSCREEN-27), dichotomized as excellent/very good and good/regular/bad; acute diabetic decompensations during the previous 3 months; mental health, dichotomized by the total SDQ score cutoff of 15; and family, classified as dysfunctional (total APGAR score≤6) or functional (total APGAR score=7-10) [32]. To assess differences between known groups, a Chi-square test was used for proportions of participants with problems, Wilcoxon nonparametric test was used for the EQ-5D-Y summary score and EQ-VAS, and unpaired *t*-test was used for KIDSCREEN scores. The magnitude of the differences between groups was assessed by the Cohen effect size (difference of mean/pooled SD) [34]. General guidelines define an effect size of 0.2 as small, 0.5 as moderate, and 0.8 as large [35]. The hypotheses raised a priori, based on available evidence, were higher EQ-5D-Y summary score (worse HRQoL) in children reporting worse general health [11], with previous acute diabetic decompensations [36,37], worse mental health [11,38], and with a dysfunctional family [39,40].

The multitrait, multimethod matrix between the EQ-5D-Y and the KIDSCREEN was constructed using Spearman correlations. The logical relationship between two instruments can be categorized as convergent and discriminant. For the convergent validity [7] (different instruments measuring a similar concept), we hypothesized substantial correlations between “mobility” (EQ-5D-Y) and “physical well-being” (KIDSCREEN-27), “feeling worried/sad/unhappy” (EQ-5D-Y) and “psychological wellbeing” (KIDSCREEN-27), and the KIDSCREEN-10 index with the EQ-5D-Y summary score and EQ-VAS. In contrast, for the discriminant validity [7] (different instruments measuring different traits or constructs), we hypothesized low correlations between the EQ-5D-Y dimension of “looking after myself” and the following three KIDSCREEN-27 dimensions: “autonomy and relationships with parents,” “social support and relationship with friends,” and “school environment.” The strength of Spearman correlations was defined [41] as insignificant (≤0.30), moderate (0.31-0.44), or substantial (0.45-0.60).

The reliability was assessed in terms of reproducibility (stability of an instrument over time) in a subsample of stable participants—those in the control group with an absolute change <4.5 points in the KIDSCREEN-10 index from the first to the fourth visit, which corresponds to a small magnitude of change (effect size<0.45). To measure the agreement in EQ-5D-Y summary score and EQ-VAS between both administrations, the intraclass correlation coefficient (ICC) was calculated. To assess responsiveness, we classified participants experiencing “improvement” as those with a change in KIDSCREEN-10 index ≥4.5 points (moderate effect size) between the first and the fourth visits. Differences for the EQ-5D-Y dimensions were compared using the McNemar paired test, while differences between the EQ-5D-Y summary score and EQ-VAS were tested with the Wilcoxon paired test. The magnitude of change was also measured by the effect size coefficient (mean of change/SD of change). Program STATA.14 software (StateCorp LP, College Station, Texas) was used in the analysis.

## Results

### Sample Characteristics

Of 205 potential candidates with T1DM, 61 were not included due to change of address, transfer to the adult unit, or no attendance at the follow-up visits; in addition, 8 subjects rejected participation. Finally, 136 participants were included in the study. Table 1 shows the characteristics of the sample. Mean age was 14 years, around half of the participants were girls, 71 (52.2%) had HbA_1c_ levels ≤7.5% (58 mmol/mL), 123 (90.4%) did not present acute diabetic decompensations during the previous 3 months, and the majority (n=95) rated their health as good or very good.

**Table 1:** Demographic and clinical characteristics of the participants (N=136).

Characteristic		Value
**Age (years)^a^, mean (SD)**		13.99 (0.2)
	8-12, n (%)	45 (33.3)
	13-15, n (%)	54 (40.0)
	16-18, n (%)	35 (25.9)
	>18, n (%)	1 (0.7)
**Sex, n (%)**		
	Girls	72 (52.9)
	Boys	64 (47.1)
**Metabolic control, HbA_1c_^b^, mean (SD)**		7.7 (1.3)
	>7.5, n (%)	65 (47.8)
	≤7.5, n (%)	71 (52.2)
**Acute diabetic decompensations (previous 3 months), n (%)**		
	Yes	13 (9.6)
	No	123 (90.4)
**General Health Question (KIDSCREEN-27), n (%)**		
	Excellent	19 (13.9)
	Very good	44 (32.4)
	Good	54 (39.7)
	Regular	17 (12.5)
	Bad	2 (1.5)
**Mental health (total SDQ^c^ score), mean (SD)**		10.64 (5.3)
	0-15, n (%)	113 (83.1)
	16-40, n (%)	23 (16.9)
**Family function (APGAR^d^), n (%)**		
	Dysfunctional	52 (38.2)
	Functional	84 (61.8)

^a^The age of one participant is missing (N=135).

^b^HbA_1c_: glycated hemoglobin.

^c^SDQ: Strengths and Difficulties Questionnaire.

^d^APGAR: Adaptation, Partnership, Growth, Affection, and Resolve.

### Distribution of Dimensions and Indices of Health-Related Quality of Life

Children and adolescents with T1DM reported relatively fewer health problems in the EQ-5D-Y dimensions (Table 2), especially for “mobility” (n=3, 2.2%) and “looking after myself” (n=4, 2.9%). Dimensions showing higher percentages of participants with problems were “having pain or discomfort” (n=47, 34.6%) and “feeling worried/sad/unhappy” (n=39, 28.6%). Means and 95% CI of KIDSCREEN-27 dimensions (Table 3) were below 50 (European sample mean [28]) for “physical well-being” (46.2, 95% CI 44.7-47.7) and over 50 for “school environment” (52.0, 95% CI 50.4-53.6) and “social support/relationship with friends” (53.6, 95% CI 52.1-55.1). As shown in Table 4, the mean (SD) of the EQ-5D-Y summary score was 8.5 (10.9), with ceiling and floor effects of 50.7% and 0%, respectively. The EQ-VAS ranged in our sample from 31 to 100, with a mean (SD) of 80.4 (14.7). The KIDSCREEN index ranged from 40.0 to 83.8, and the mean (SD) was 49.7 (8.3).

**Table 2:** Distribution of the EuroQol-5D-Youth (EQ-5D-Y) dimensions.

EQ-5D-Y dimensions	No problems, n (%)	Some problems, n (%)	A lot of problems, n (%)
Mobility	133 (97.8)	3 (2.2)	0 (0.0)
Looking after myself	132 (97.1)	4 (2.9)	0 (0.0)
Doing usual activities	119 (87.5)	17 (12.5)	0 (0.0)
Having pain/discomfort	89 (65.4)	45 (33.1)	2 (1.5)
Feeling worried/sad/unhappy	97 (71.3)	35 (25.7)	4 (2.9)

**Table 3:** Distribution of KIDSCREEN-27 dimensions.

KIDSCREEN-27 dimensions	Mean (SD), 95% CI	Median
Physical well-being	46.2 (9.0), 44.7-47.7	44.7
Autonomy/relationships with parents	50.5 (8.2), 49.1-51.9	49.5
School environment	52.0 (9.3), 50.4-53.6	51.1
Social support and relationship with friends	53.6 (9.1), 52.1-55.1	53.3
Psychological well-being	49.7 (9.7), 48.1-51.3	48.5

**Table 4:** Distribution of the EuroQol-5D-Youth (EQ-5D-Y) summary score, EuroQol-Visual Analog Scale (EQ-VAS), and KIDSCREEN-10 index.

Distribution of scores	EQ-5D-Y summary score	EQ-VAS	KIDSCREEN-10 index
Theoretical range	0-100	0-100	–3.54 to 83,81
Observed range	0-40	31-100	40.0-83.81
Proportion of participants with the best health (%)	50.7	8.1	0.7
Proportion of participants with the worst health (%)	0	0	0
Mean (SD)	8.5 (10.9)	80.4 (14.7)	49.7 (8.3)

### Construct Validity of the EuroQol-5D-Youth Online

Table 5 shows statistically significant HRQoL differences between groups defined by their general health in three of five EQ-5D-Y dimensions as well as EQ-5D-Y summary score, EQ-VAS, and KIDSCREEN-10 index (*P<*.001, *P<*.001, and *P*=.02, respectively) with moderate-large effect sizes (0.7, 1.0, and 0.6, respectively). Similar significant HRQoL differences were found according to mental health with large effect sizes, except for the EQ-VAS. The only significant difference between those with and without acute diabetic decompensations was found in the “looking after myself” dimension (*P*=.005). Finally, HRQoL differences between functional and dysfunctional families were statistically significant for the “having pain and discomfort” EQ-5D-Y dimension (*P*=.03) and KIDSCREEN-10 index (*P*<.001).

**Table 5:** Comparison of health-related quality of life between known groups measured at baseline.

Variables		EQ-5D-Y^a^ dimensions					EQ-5D-Y summary score, mean (SD), median	EQ-VAS^b^, mean (SD), median	KIDSCREEN-10 index, mean (SD), median
		M^c^	LAM^d^	DUA^e^	HP/D^f^	W/S/U^g^			
**General health question**									
	Excellent/very good	0.0%	3.2%	4.8%	22.2%	19.1%	5.08 (8.4), 0	87.6 (10.5), 90	52.24 (6.9), 53.1
	Good/regular/bad	4.1%	2.7%	19.2%	45.2%	37.0%	11.51 (12.1), 10	74.1 (15.0), 75	47.47 (8.9), 45.7
	*P* value	.25	1.00	.02^h^	<.01^h^	.02^h^	<.001^h^	<.001^h^	.02^h^
	Effect size (95% CI)	N/A^i^	N/A	N/A	N/A	N/A	0.7 (0.39-1.09)	1.0 (0.66-1.38)	0.6 (0.25-0.94)
**Acute diabetic decompensations**									
	Yes	7.7%	15.4%	23.1%	38.5%	46.2%	13.85 (13.2), 10.0	74.9 (18.0), 75	50.21 (9.8), 46.9
	No	1.6%	1.6%	11.4%	34.2%	26.8%	7.97 (10.6), 0	80.9 (14.3), 81	49.63 (8.2), 48.3
	*P* value	.26	.005^h^	.23	.76	.14	.08	.21	.81
	Effect size (95% CI)	N/A	N/A	N/A	N/A	N/A	0.5 (–0.04 to 1.12)	0.4 (–0.17 to 0.99)	0.07 (–0.5 to 0.65)
**Mental health (SDQ^j^)**									
	Score 0-15	0.9%	2.7%	8.9%	28.3%	20.4%	6.11 (8.4), 0	81.3 (14.8), 81	50.84 (8.3), 49.8
	Score 16-40	8.7%	4.4%	30.4%	65.2%	69.6%	20.43 (14.3), 20	75.7 (13.5), 75	44.01 (5.6), 43.4
	*P* value	.07	.53	.004^h^	.001^h^	<.001^h^	<.001^h^	.06	<.001^h^
	Effect size (95% CI)	N/A	N/A	N/A	N/A	N/A	1.5 (1.01-1.98)	0.4 (–0.07 to 0.83)	0.9 (0.40-1.32)
**Family function (APGAR** ^k^ **)**									
	Score≤6	1.2%	4.8%	10.7%	27.4%	25.0%	7.02 (9.9), 0	81.6 (15.1), 85	51.46 (8.6), 50.6
	Score 7-10	3.9%	0.0%	15.4%	46.2%	34.6%	10.96 (12.3), 10	78.3 (13.9), 80	46.81 (6.9), 46.9
	*P* value	.56	.30	.42	.03^h^	.23	.06	.12	<.001^h^
	Effect size (95% CI)	N/A	N/A	N/A	N/A	N/A	0.4 (0.01-0.71)	0.2 (–0.12 to 0.58)	0.6 (0.22-0.93)

^a^EQ-5D-Y: EuroQol-5D-Youth.

^b^EQ-VAS: EuroQol-Visual Analog Scale.

^c^M: mobility.

^d^LAM: looking after myself.

^e^DUA: doing usual activities.

^f^HP/D: having pain/discomfort.

^g^W/S/U: feeling worried/sad/unhappy.

^h^Statistically significant difference.

^i^N/A: not applicable.

^j^SDQ: Strengths and Difficulties Questionnaire.

^k^APGAR: Adaptation, Partnership, Growth, Affection, and Resolve.

Table 6 presents the multitrait, multimethod matrix between EQ-5D-Y and KIDSCREEN. Regarding convergent validity, of the four correlations previously hypothesized as substantial, subscript a), three obtained coefficient values of 0.45 or greater: 0.58 between EQ-5D-Y “feeling worried/sad/unhappy” and KIDSCREEN “psychological wellbeing,” 0.50 between the EQ-5D-Y summary score and KIDSCREEN-10 index, and 0.46 between the EQ-VAS and KIDSCREEN-10 index. In contrast, the correlation between the mobility dimension of the EQ-5D-Y and the physical well-being dimension of the KIDSCREEN-27 was 0.21. For discriminant validity, the three relationships hypothesized as insignificant (subscript b) ranged from 0.02 to 0.06, as expected.

**Table 6:** Multitrait, multimethod matrix between the EuroQol-5D-Youth (EQ-5D-Y) and the KIDSCREEN in children and adolescents with type 1 diabetes mellitus at baseline evaluation.

EQ-5D-Y	KIDSCREEN-27					KIDSCREEN-10 index
	Physical well-being	Psychological well-being	Autonomy and relationships with parents	Social support and relationship with friends	School environment	
Mobility	0.21^a^	0.19	0.02	0.06	0.12	0.16
Looking after myself	0.02	0.07	0.06^b^	0.02^b^	0.06^b^	0.03
Doing usual activities	0.17	0.29	0.08	0.14	0.25	0.25
Having pain/discomfort	0.27	0.31	0.23	0.13	0.17	0.32
Feeling worried/sad/unhappy	0.30	0.58^a^	0.22	0.36	0.40	0.56
EQ-5D-Y summary score	0.23	0.10	0.01	0.18	0.00	0.50^a^
EuroQol-Visual Analog Scale	0.38	0.31	0.21	0.14	0.35	0.46^a^

^a^Correlations hypothesized as substantial (0.45-0.60).

^b^Correlations hypothesized as insignificant (≤0.30).

### Reproducibility and Responsiveness

Table 7 shows EQ-5D-Y test-retest results in the “stable” subsample between the first and the fourth visit. The ICC was 0.83 and 0.74 for the EQ-5D-Y summary score and EQ-VAS, demonstrating stability over time for this subsample. Regarding responsiveness, this “improvement” subsample presented significant changes in three dimensions, in the EQ-5D-Y summary score, and EQ-VAS between the first visit and the fourth visit. The effect sizes were 0.70 and 0.74, indicating an improvement of moderate-to-large magnitude.

**Table 7:** Reproducibility and responsiveness of EuroQol-5D-Youth (EQ-5D-Y): problems reported by each dimension, summary score, and EuroQol-Visual Analog Scale (EQ-VAS) at the first and fourth visits.

EQ-5D-Y		Test-retest reproducibility among stable participants (n=18)			Responsiveness among participants with improvement (n=58)		
		First visit	Fourth visit	*P* value	First visit	Fourth visit	*P* value
**EQ-5D-Y dimensions**							
	Mobility, n (%)	0 (0.0)	1 (5.6)	—^a^	3 (5.2)	1 (1.7)	.63
	Looking after myself, n (%)	1 (5.6)	0 (0.0)	—	2 (3.5)	1 (1.7)	>.99
	Doing usual activities, n (%)	1 (5.6)	1 (5.6)	.06	9 (15.5)	2 (3.5)	.02^b^
	Having pain/discomfort, n (%)	4 (22.2)	4 (22.2)	.13	27 (46.6)	11 (18.9)	<.001^b^
	Feeling worried/sad/unhappy, n (%)	6 (33.3)	5 (27.8)	.14	23 (39.7)	10 (17.2)	<.001^b^
**EQ-5D-Y summary score**				.71			<.001^b^
	Mean (SD)	6.67 (9.1)	6.11 (7.8)		11.90 (12.6)	4.31(7.0)	
	Effect size (95% CI)	N/A^c^	0.07 (–0.61 to 0.74)		N/A	0.70 (0.31 to 1.02)	
	ICC^d^ (95% CI)	N/A	0.83 (0.55 to 0.94)		N/A	N/A	
**EQ-VAS**				.15			<.001^b^
	Mean (SD)	82 (13.5)	86 (9.1)		76.0 (16.6)	86.9 (13.0)	
	Effect size (95% CI)	N/A	0.35 (–0.34 to 1.03)		N/A	0.74 (0.36 to 1.12)	
	ICC (95% CI)	N/A	0.74 (0.31 to 0.90)		N/A	N/A	

^a^Not available.

^b^Statistically significant difference.

^c^N/A: not applicable.

^d^ICC: intraclass correlation coefficient.

## Discussion

To the best of our knowledge, this is the first study evaluating metric properties of the new EQ-5D-Y in T1DM children and adolescents. We found this generic preference-based instrument to be acceptable for children and adolescents with T1DM and easy to administer online, but with a high ceiling effect (50.7% of participants reported no problem in any dimension). The EQ-5D-Y showed good validity, considering the results obtained in the known groups’ analysis based on general health, acute diabetic decompensations, mental health, and family function, and those obtained from the multitrait, multimethod matrix with KIDSCREEN. Test-retest reproducibility was high, indicating good reliability, and the moderate-large change observed between the first and fourth visits among the selected subsample of “improvement” supports its responsiveness.

Of the 136 participants in the T1DM study, all responded to the EQ-5D-Y at the first visit and 119 (87.5%) responded at the fourth visit, which is similar to the response rates reported by studies carried out in the clinical (96% [18]) and school (77% [16]) settings. It is important to remark that the participants answering the EQ-5D-Y entirely completed the instrument, including the five dimensions, and the EQ-VAS. These rates of completion support the acceptability of the EQ-5D-Y among children and adolescents with T1DM when administered online. Taking into account the quick expansion of online recreational habits among the new generations born in the digital era, acceptability could be even higher with the incorporation of other devices such as apps for smartphones or tablets. However, completion rates in routine clinical practice could be lower than under clinical trial conditions, which indicates the need for closer monitorization.

Ceiling effect in the sample of children and adolescents with T1DM was high for the EQ-5D-Y summary score (50.7%) and for the three out of its five dimensions, which exceeded 85% of participants reporting no problem (“mobility,” “looking after myself,” and “doing usual activities”). A European multicountry study of EQ-5D-Y metric properties [11] reported a similar ceiling effect for these dimensions, which was up to 80% in children with chronic conditions. Half of the children and adolescents with no problem in any dimension (value=0 in EQ-5D-Y summary score) could be considered to show a really high ceiling effect, taking into account the established recommendations of 15% for HRQoL scores [42]. However, this is a general standard, as there are none specific for children. This high ceiling effect could be considered coherent with the relative low impact of diabetes on HRQoL for most children and adolescents, partially explained by their capacity of adaptation to the demanding tasks related to care [43]. The main disadvantage of this high ceiling effect is that it avoids the detection of improvement in children and adolescents who reported the highest level of health, which could affect the instrument’s responsiveness.

On the other hand, our study showed a substantial proportion of children and adolescents with T1DM reporting problems in the dimensions of “having pain or discomfort” (n=47, 34.6%) and “feeling worried/sad/unhappy” (n=43, 28.7%). These results are consistent with a previous study [44] showing this latter EQ-5D-Y dimension as the most affected in children and adolescents with T1DM. They are also consistent with studies using the 21-item Beck Depression Inventory and the Child Health Questionnaire PF-50 [45,46], which highlighted the impact of T1DM on the psychological dimension, especially due to the lack of autonomy, worrying for future chronic complications and the self-care routines that these children and adolescents have to experience.

As hypothesized, the EQ-5D-Y presented a good discrimination capacity between groups defined by the general health question and SDQ score in the summary score and in three dimensions: “doing usual activities,” “having pain/discomfort,” and “feeling worried/sad/unhappy.” These dimensions presented the highest correlations with general and mental health in previous studies of children and adolescents with T1DM [41] or chronic conditions [10]. The magnitude of the difference between groups defined by these constructs was larger when HRQoL was measured with the EQ-5D-Y summary score (effect size for general health and mental health groups=0.7 and 1.5, respectively) than with the KIDSCREEN-10 index (effect size for general health and mental health groups=0.6 and 0.9, respectively).

It is important to highlight the problems reported in the “looking after myself” dimension among those participants who had previously experienced acute diabetic decompensations, which is consistent with previous studies comparing good and bad metabolic control [37,47]. These participants showed differences in the physical well-being and health perception dimensions, measured with KINDL-R [37] and the Diabetes Quality of Life questionnaire [42]. A priori hypotheses on the relationship between family function and HRQoL were based on studies that reported an increased risk for depressive symptoms in families with a disadvantaged socioeconomic status [39,48]. Our results, showing problems of “having pain and discomfort” more frequently and poorer HRQoL with KIDSCREEN-10 index in children and adolescents with dysfunctional families, are in line with the social component of health [39,48].

Correlations between EQ-5D-Y and KIDSCREEN showed the expected pattern for convergent and divergent validity, which were very similar to those obtained in the abovementioned European multicountry study [11]. The dimensions reflecting the emotional aspects of EQ-5D-Y (“feeling worried/sad/unhappy”) and KIDSCREEN-27 (“psychological well-being”), in addition to the EQ-5D-Y summary score and EQ-VAS, presented a substantial correlation with the KIDSCREEN-10 index, which provides a summary of the HRQoL. The EQ-5D-Y “mobility” dimension failed to display convergent validity with the KIDSCREEN dimension “physical wellbeing,” similar to a previous study [11] (*r*=0.10-0.25). Considering the content of the instruments, it can be argued that KIDSCREEN is more focused on the energy level and less focused on the physical functioning, which is the case of the EQ-5D-Y.

Our study shows good reproducibility of the questionnaire according to the established standards [7] for reliability coefficients: 0.7 and 0.9 for group and individual measurements, respectively. The ICC of 0.83 and 0.74 obtained for the EQ-5D-Y summary score and EQ-VAS allows recommending its use. Nine months between the first and the fourth visit is too long a period to evaluate reproducibility, since HRQoL could change considerably during this time. This long period explains the low number of children and adolescents in the stable subsample (n=18). Previous EQ-5D-Y studies used the kappa coefficient to evaluate test-retest reporting from good to fair agreement, according to dimensions [11,15].

The EQ-5D-Y detected differences in the selected subsample of children and adolescents with T1DM classified as experiencing “improvement” between the first and the fourth visit, showing the capacity of this instrument to detect change. It is important to highlight that no other studies testing the responsiveness of the EQ-5D-Y have been found. A systematic review assessing metric properties of diabetes-specific HRQoL instruments also highlighted that the lack of testing for responsiveness is a major shortcoming [49]. The EQ-5D-Y summary score indicates a moderate-large improvement over time, a magnitude which can be considered reasonable when accounting for the length of the follow-up of participants (9 months), the nature of the intervention, and the course of the T1DM.

The T1DM clinical trial provides a comprehensive and complete database of EQ-5D-Y and disease-related variables to allow assessment of metric properties including validity, reliability, and responsiveness. However, some limitations need to be addressed. First, the assessment of the acceptability is not complete: Other indicators such as administration time needed to complete the questionnaire and patient views about the instrument should be included in further studies. Second, since the EuroQol group has not developed the value set for the EQ-5D-Y yet [23], the unweighted summary score was calculated as was done in previous studies [24-26]. However, this summary score did not allow cost-utility analysis. Third, the EQ-5D-Y does not cover social dimension, a key aspect of children’s HRQoL, but it presents an acceptable capacity of discrimination between functional and dysfunctional families in our study. There are other generic pediatric instruments that cover social dimensions, such as KIDSCREEN [50] (“autonomy and relationships with parents,” “social support,” “relationship with friends,” and “school environment”), PedsQol [51] (“social functioning” and “school functioning”), or the Child Health Questionnaire [52] (“role/social emotional and behavioral functioning”). Fourth, this is a secondary analysis of a study designed for purposes other than the evaluation of the metric properties of EQ-5D-Y. For example, as commented above, the 9-month period between test and retest is too long for measuring reproducibility, and no large change is expected with the intervention applied. Furthermore, results on stability should be interpreted with caution due to the small sample size. Finally, regarding external validity, the results of this study should not be generalized to other chronic conditions.

Despite the limitations discussed above, our results provide considerable evidence supporting the appropriate metric properties of the EQ-5D-Y in patients with T1DM. In conclusion, these findings suggest that the EQ-5D-Y administered online is an acceptable, valid, reliable, and responsive instrument for evaluating HRQoL in children and adolescents with this chronic condition. Since it is a preference-based health status measure, it will allow calculating quality-adjusted life-years (combining both the quantity and quality of life) for economic evaluations, once social preferences specifically for children become available. Given its short and easy administration, the EQ-5D-Y is a practical instrument to implement in primary care to routine monitoring. It is a promising instrument to compare the efficiency of different programs or treatment strategies, helping prioritize and invest at different levels.

## Abbreviations

APGAR: Adaptation, Partnership, Growth, Affection, and Resolve
DUA: doing usual activities
EQ-5D-Y: Youth EuroQoL version
EQ-VAS: EuroQol-Visual Analog Scale
EQ: EuroQol
ES: effect size
HbA_1c_: glycated hemoglobin
HP/D: having pain/discomfort
HRQoL: health-related quality of life
ICC: intraclass correlation coefficient
LAM: looking after myself
SDQ: Strengths and Difficulties Questionnaire
T1DM: type I diabetes mellitus
W/S/U: feeling worried/sad/unhappy

## References

[1] Kalyva E, Malakonaki E, Eiser C, Mamoulakis D (2011). Health-related quality of life (HRQoL) of children with type 1 diabetes mellitus (T1DM): self and parental perceptions. Pediatr Diabetes.

[2] American Diabetes Association (2019). 13. Children and Adolescents: Standards of Medical Care in Diabetes-2019. Diabetes Care.

[3] Mcdowell I (2019). Factors affecting quality of life in lower limb amputees. Measuring Health: A Guide To Rating Scales And Questionnaires.

[4] Guyatt GH, Feeny DH, Patrick DL (1993). Measuring health-related quality of life. Ann Intern Med.

[5] Debono M, Cachia E (2007). The impact of diabetes on psychological well being and quality of life. The role of patient education. Psychol Health Med.

[6] van Reenen M, Janssen B, Oppe M, Kreimeier S, Greiner W (2014). EuroQol.

[7] Aaronson N, Alonso J, Burnam A, Lohr KN, Patrick DL, Perrin E, Stein RE (2002). Assessing health status and quality-of-life instruments: attributes and review criteria. Qual Life Res.

[8] Eiser C, Morse R (2001). A review of measures of quality of life for children with chronic illness. Arch Dis Child.

[9] Gusi N, Perez-Sousa MA, Gozalo-Delgado M, Olivares PR (2014). Validity and reliability of the spanish EQ-5D-Y proxy version. An Pediatr (Barc).

[10] Raat H, Bonsel GJ, Hoogeveen WC, Essink-Bot M, Dutch HUI Group (2004). Feasibility and reliability of a mailed questionnaire to obtain visual analogue scale valuations for health states defined by the Health Utilities Index Mark 3. Med Care.

[11] Ravens-Sieberer U, Wille N, Badia X, Bonsel G, Burström K, Cavrini G, Devlin N, Egmar A, Gusi N, Herdman M, Jelsma J, Kind P, Olivares PR, Scalone L, Greiner W (2010). Feasibility, reliability, and validity of the EQ-5D-Y: results from a multinational study. Qual Life Res.

[12] Burström K, Svartengren M, Egmar A (2011). Testing a Swedish child-friendly pilot version of the EQ-5D instrument--initial results. Eur J Public Health.

[13] Jelsma J (2010). A comparison of the performance of the EQ-5D and the EQ-5D-Y health-related quality of life instruments in South African children. Int J Rehabil Res.

[14] Jelsma J, Ramma L (2010). How do children at special schools and their parents perceive their HRQoL compared to children at open schools?. Health Qual Life Outcomes.

[15] Scott D, Ferguson GD, Jelsma J (2017). The use of the EQ-5D-Y health related quality of life outcome measure in children in the Western Cape, South Africa: psychometric properties, feasibility and usefulness - a longitudinal, analytical study. Health Qual Life Outcomes.

[16] Robles N, Rajmil L, Rodriguez-Arjona D, Azuara M, Codina F, Raat H, Ravens-Sieberer U, Herdman M (2015). Development of the web-based Spanish and Catalan versions of the Euroqol 5D-Y (EQ-5D-Y) and comparison of results with the paper version. Health Qual Life Outcomes.

[17] Eidt-Koch D, Mittendorf T, Greiner W (2009). Cross-sectional validity of the EQ-5D-Y as a generic health outcome instrument in children and adolescents with cystic fibrosis in Germany. BMC Pediatr.

[18] Bergfors Sofi, Åström Mimmi, Burström Kristina, Egmar Ann-Charlotte (2015). Measuring health-related quality of life with the EQ-5D-Y instrument in children and adolescents with asthma. Acta Paediatr.

[19] Hsu Chien-Ning, Lin Hsiang-Wen, Pickard A Simon, Tain You-Lin (2018). EQ-5D-Y for the assessment of health-related quality of life among Taiwanese youth with mild-to-moderate chronic kidney disease. Int J Qual Health Care.

[20] Miranda Velasco MJ, Domínguez Martín E, Arroyo Díez F J, Méndez Pérez P, González de Buitrago Amigo J (2012). Health related quality of life in type 1 diabetes mellitus. An Pediatr (Barc).

[21] Murillo M, Bel J, Pérez J, Corripio R, Carreras G, Herrero X, Mengibar J, Rodriguez-Arjona D, Ravens-Sieberer U, Raat H, Rajmil L (2017). Health-related quality of life (HRQOL) and its associated factors in children with Type 1 Diabetes Mellitus (T1DM). BMC Pediatr.

[22] Murillo M, Bel J, Pérez J, Corripio R, Carreras G, Herrero X, Mengibar J, Rodriguez-Arjona D, Ravens-Sieberer U, Raat H, Rajmil L (2017). Impact of monitoring health-related quality of life in clinical practice in children with type 1 diabetes mellitus. Qual Life Res.

[23] Kreimeier S, Greiner W (2019). EQ-5D-Y as a Health-Related Quality of Life Instrument for Children and Adolescents: The Instrument's Characteristics, Development, Current Use, and Challenges of Developing Its Value Set. Value Health.

[24] Wilke CT, Pickard AS, Walton SM, Moock J, Kohlmann T, Lee TA (2010). Statistical implications of utility weighted and equally weighted HRQL measures: an empirical study. Health Econ.

[25] Prieto L, Sacristán JA (2004). What is the value of social values? The uselessness of assessing health-related quality of life through preference measures. BMC Med Res Methodol.

[26] Hinz A, Kohlmann T, Stöbel-Richter Y, Zenger M, Brähler E (2014). The quality of life questionnaire EQ-5D-5L: psychometric properties and normative values for the general German population. Qual Life Res.

[27] Ravens-Sieberer U, Erhart M, Rajmil L, Herdman M, Auquier P, Bruil J, Power M, Duer W, Abel T, Czemy L, Mazur J, Czimbalmos A, Tountas Y, Hagquist C, Kilroe J, European KIDSCREEN Group (2010). Reliability, construct and criterion validity of the KIDSCREEN-10 score: a short measure for children and adolescents' well-being and health-related quality of life. Qual Life Res.

[28] Ravens-Sieberer U, Auquier P, Erhart M, Gosch A, Rajmil L, Bruil J, Power M, Duer W, Cloetta B, Czemy L, Mazur J, Czimbalmos A, Tountas Y, Hagquist C, Kilroe J, European KIDSCREEN Group (2007). The KIDSCREEN-27 quality of life measure for children and adolescents: psychometric results from a cross-cultural survey in 13 European countries. Qual Life Res.

[29] Goodman R (1997). The Strengths and Difficulties Questionnaire: a research note. J Child Psychol Psychiatry.

[30] Cladellas Y (2007). Dep Psiquiatr i Med Leg Fac Med.

[31] Lima Rodríguez JS, Lima Serrano M, Jiménez Picón N, Domínguez Sánchez I (2012). Reliability and construct validity of an instrument to asses the Self-perception of Family Health Status [in Spanish]. Rev Esp Salud Publica.

[32] Smilkstein G, Ashworth C, Montano D (1982). Validity and reliability of the family APGAR as a test of family function. J Fam Pract.

[33] Rosner B (2019). Fundamentals of Biostatistics.

[34] Sullivan GM, Feinn R (2012). Using Effect Size-or Why the P Value Is Not Enough. J Grad Med Educ.

[35] Cohen J (1988). Statistical power analysis for the behavioral sciences.

[36] Davis RE, Morrissey M, Peters JR, Wittrup-Jensen K, Kennedy-Martin T, Currie CJ (2005). Impact of hypoglycaemia on quality of life and productivity in type 1 and type 2 diabetes. Curr Med Res Opin.

[37] Wagner VM, Müller-Godeffroy E, von Sengbusch S, Häger S, Thyen U (2005). Age, metabolic control and type of insulin regime influences health-related quality of life in children and adolescents with type 1 diabetes mellitus. Eur J Pediatr.

[38] Kristensen LJ, Birkebaek NH, Mose AH, Hohwü L, Thastum M (2014). Symptoms of emotional, behavioral, and social difficulties in the danish population of children and adolescents with type 1 diabetes--results of a national survey. PLoS One.

[39] Delamater AM, Shaw KH, Applegate EB, Pratt IA, Eidson M, Lancelotta GX, Gonzalez-Mendoza L, Richton S (1999). Risk for metabolic control problems in minority youth with diabetes. Diabetes Care.

[40] Silverstein J, Cheng P, Ruedy KJ, Kollman C, Beck RW, Klingensmith GJ, Wood JR, Willi S, Bacha F, Lee J, Cengiz E, Redondo MJ, Tamborlane WV, Pediatric Diabetes Consortium (2015). Depressive Symptoms in Youth With Type 1 or Type 2 Diabetes: Results of the Pediatric Diabetes Consortium Screening Assessment of Depression in Diabetes Study. Diabetes Care.

[41] Burnand B, Kernan WN, Feinstein AR (1990). Indexes and boundaries for. J Clin Epidemiol.

[42] Terwee CB, Bot SDM, de Boer Michael R, van der Windt Daniëlle A W M, Knol DL, Dekker J, Bouter LM, de Vet Henrica C W (2007). Quality criteria were proposed for measurement properties of health status questionnaires. J Clin Epidemiol.

[43] Sprangers MA, Schwartz CE (1999). Integrating response shift into health-related quality of life research: a theoretical model. Soc Sci Med.

[44] Lustman PJ, Freedland KE, Carney RM, Hong BA, Clouse RE (1992). Similarity of depression in diabetic and psychiatric patients. Psychosom Med.

[45] Wake M, Hesketh K, Cameron F (2000). The Child Health Questionnaire in children with diabetes: cross-sectional survey of parent and adolescent-reported functional health status. Diabet Med.

[46] Zenlea IS, Mednick L, Rein J, Quinn M, Wolfsdorf J, Rhodes ET (2014). Routine behavioral and mental health screening in young children with type 1 diabetes mellitus. Pediatr Diabetes.

[47] Hoey H, Aanstoot HJ, Chiarelli F, Daneman D, Danne T, Dorchy H, Fitzgerald M, Garandeau P, Greene S, Holl R, Hougaard P, Kaprio E, Kocova M, Lynggaard H, Martul P, Matsuura N, McGee HM, Mortensen HB, Robertson K, Schoenle E, Sovik O, Swift P, Tsou RM, Vanelli M, Aman J (2001). Good metabolic control is associated with better quality of life in 2,101 adolescents with type 1 diabetes. Diabetes Care.

[48] Ismail H (2011). Self-rated health and factors influencing responses among young Egyptian type 1 diabetes patients. BMC Public Health.

[49] Garratt AM, Schmidt L, Fitzpatrick R (2002). Patient-assessed health outcome measures for diabetes: a structured review. Diabet Med.

[50] Ravens-Sieberer U, Herdman M, Devine J, Otto C, Bullinger M, Rose M, Klasen F (2014). The European KIDSCREEN approach to measure quality of life and well-being in children: development, current application, and future advances. Qual Life Res.

[51] Desai AD, Zhou C, Stanford S, Haaland W, Varni JW, Mangione-Smith RM (2014). Validity and responsiveness of the pediatric quality of life inventory (PedsQL) 4.0 generic core scales in the pediatric inpatient setting. JAMA Pediatr.

[52] Landgraf JM, van Grieken A, Raat H (2018). Giving voice to the child perspective: psychometrics and relative precision findings for the Child Health Questionnaire self-report short form (CHQ-CF45). Qual Life Res.

